# Physical activity and dietary behaviors: a phenomenological analysis of experiences of Ghanaians during the COVID-19 lockdown

**DOI:** 10.11604/pamj.2020.37.199.23733

**Published:** 2020-10-29

**Authors:** Edward Wilson Ansah, Jacob Owusu Sarfo, Daniel Apaak

**Affiliations:** 1 Department of Health, Physical Education and Recreation, University of Cape Coast, Cape Coast, Ghana

**Keywords:** COVID-19 lockdown, Ghana, physical activity, weight gain, boredom, dietary habits

## Abstract

**Introduction:**

the COVID-19 pandemic has necessitated many public health preventive measures including lockdowns or curfews. However, because humans are used to working and moving up and down, they would need to find ways to avert the negatives associated with the COVID-19 induced lockdown. Therefore, the purpose of this study was to qualitatively explore experiences of Ghanaians during the lockdown period in terms of physical activity, dietary behaviors, boredom, and changes in weight.

**Methods:**

using a phenomenological approach, we analyzed data from 27 persons from Accra, Tema, and Kumasi who filled our online open-ended survey. We created open-ended items and circulated online (between 21^st^ April, 2020 and 10^th^ May, 2020) to persons who experienced the lockdown. We analyzed the data using Colaizzi´s 7-step phenomenological approach.

**Results:**

many people felt very bored and frustrated during the lockdown period, and some of these people resorted to physical exercise routines either individually or collectively as family. However, many experienced tremendous physical inactivity because of lack of space. They experienced poor eating behaviors, all of which resulted in reported weight gains.

**Conclusion:**

the COVID-19 pandemic lockdown has caused boredom and frustrations to quite a number of people. Physical inactivity increased because of lack of space, coupled with poor eating habits producing high levels of weight gain among people who experienced the lockdown in Ghana. There is therefore an urgent need to teach these people how to exercise within limited space and how to eat healthily during times of restriction.

## Introduction

The world is overwhelmed with COVID-19 pandemic, halting national and global health systems. The devastations of the pandemic are resulting in significant number of fatalities in places such as the United States of America (USA), Europe, and the United Kingdom [1]. Though it is difficult to estimate the numbers in cases, deaths and recoveries [2], the effects of the COVID-19 pandemic extend beyond health, economy, socio-political, education, and religion, to social interactions, and psycho-emotional dimensions of individuals, communities, and nations [3]. The COVID-19 pandemic is equally causing its devastation on the African continent like other continents. South Africa has recorded the highest number of cases with Ghana being among the top-five most hit nations in the continent [1]. Ghana recorded its first case, an imported case, of the virus on the 12^th^ of March, 2020, and since then, the country is steadily recording increasing numbers which stand at 5638, with 28 deaths and 1460 recoveries as of 15^th^ May, 2020 [4].

The pandemic has caused governments all over the world including Ghana´s government to initiate series of public health preventive measures (PHPM) such as closure of all educational institutions, religious activities, advocacy on handwashing with soap under running water, frequent use of alcohol-based hand sanitizer, “stay at home” policy, socio-physical distancing, lockdown, and recently the use of nose-mouth masks especially in public places [5, 6]. However, Ghanaian society is collectivistic in nature, where intrapersonal interactions are needed culturally for the day-to-day life of its citizens. Therefore, many of these COVID-19 pandemic-induced restrictions are alien and new to most Ghanaians. As such, national measures to impose citizenry movement restrictions like lockdowns have a great propensity to create confusion on how to manage life including happy living, being active, eating healthy, and preventing unnecessary weight gain [7]. Measures like the lockdown, “stay at home”, working from home, and many other restrictive movements yield sedentariness, that is, doing low energy expenditure work from sitting or resting positions [7]. Thus, individuals who experienced the lockdown in Accra, Tema, and Greater Kumasi may also be experiencing deteriorating well-being and quality of life [8]. Besides, such a period may have a detrimental influence on physical activity (PA) levels, eating behavior, and boredom [7], three “masters” to weight gain. Therefore, there must be a concern about how individuals dealt with their sedentariness during this period of lockdown since sedentary behavior increased during the “stay at home” and lockdown times, induced by the COVID-19 [6, 9].

Furthermore, the adverse health effects of physical inactivity are established [10, 11]. Physical inactivity or sedentary lifestyle is an underlining factor in the development of illnesses like diabetes Type 2, some cancers, cardiovascular disease, hypertension, and osteoarthritis- which are directly related to decreasing longevity and quality of life [12]. Recently, some reports have called for increased PA during this period of COVID-19 induced restricted movement [7, 13]. The call for increase in PA aims to prevent COVID-19 induced chronic illnesses such as obesity, hypertension, diabetes, cardiopulmonary, and musculoskeletal disorders [14, 15], and psychological health conditions including stress, and well-being, and other mental health effects [16]. This is because COVID-19 and its PHPMs have created an obesogenic environment fertile for sedentary life [12]. However, it is not clear and documented how individuals´ in Accra, Tema, and Greater Kumasi dealt with their state of PA levels, eating behavior, and increased boredom during the period of lockdown. Therefore, this study aimed to qualitatively explore the experiences of city dwellers in Accra, Tema, and Greater Kumasi who experienced the 21-day lockdown as COVID-19 pandemic containment measure in Ghana.

## Methods

Using an empirical Colaizzi's [17] phenomenological approach, we obtained detailed descriptions of the experiences (i.e. boredom, PA, and inactivity behavior, eating habit, and change in weight) of individuals who had experienced the COVID-19 pandemic lockdown in the cities of Accra, Tema, and Greater Kumasi in Ghana. The focus of phenomenological research is to describe the commonalities of experiences across a particular population [18]. Colaizzi's [17] phenomenological approach focuses on the experiences and observes shared occurrences among research participants. Accordingly, the “approach guarantees the authenticity of the collected experience of participants to adhere to scientific standards” [19]. The participants were purposively sampled for this study due to their diverse backgrounds to share their experiences, using different social media platforms.

We created online semi-structured open-ended questions soliciting from the participants how they experienced the COVID-19 lockdown in terms of boredom, PA engagement, sedentary life, and eating habits. The instrument also collected participants´ background information such as gender, age, educational level, type of employment, and the number of days spent under the lockdown. The open-ended questions included items like:

*“What are some of your experiences regarding physical activity or sedentary lifestyle (inactivity) at home during the lockdown?”, “Tell us about your eating habits during the lockdown period”, “Tell us about how the lockdown created boredom for you at home”, “Explain to us how the lockdown has affected your weight, you gain or not”*.

This semi-structured open-ended instrument was created with Google Form and circulated on social media platforms such as WhatsApp and Facebook from 21^st^ April, 2020 to 10^th^ May, 2020. The introductory part to the instrument contained information such as the purpose of the research, voluntary participation of participants and confidentiality of their information, potential risk involved, and their consent to take part in the study, and that only individuals who were in the lockdown cities, Accra, Tema, and the Greater Kumasi, Ghana were to fill the instrument. Thus, participants sat at their convenience and filled the instrument, and they received no tangible rewards. We submitted the instrument to experts (a Professor in Sports Psychology and a Senior Lecturer in Physical Education) who scrutinized the items for validity. Also, the Institutional Review Board of the University of Cape Coast approved the research. The project was conducted following the principles of the declaration of Helsinki for human research (6^th^ revision, 2008).

We adopted Colaizzi´s [17] phenomenological method to analyze the data i.e. the transcripts. The analysis involved reading the transcript several times to understand the meanings expressed, identifying significant phrases, and restating them in general terms. These processes helped to formulate meanings from the transcripts and validate them via research team discussions to reach consensus. We further identified and organized themes into sub-themes and developed a comprehensive description of themes. We employed several strategies to increase trustworthiness, and credibility of the results and findings of the study. Credibility was achieved when participants at their convenience and on their own had written/typed their experiences (responses) regarding PA, eating habits, feeling of boredom, and weight gain as experienced during the COVID-19 lockdown in Ghana. Moreover, one of the co-authors independently analyzed the transcripts by bracketing data on preconceived ideas and following strictly the Colaizzi´s approach. The findings were then sent to the two other authors who jointly cross-checked, discussed, and agreed on themes and sub-themes. However, where there were unresolved disagreements, they invited the other author (who did the initial analysis) for his opinion. Meanwhile, we contracted a qualitative expert in Health Promotion who also cross-checked and validated our themes and sub-themes based on the transcripts.

## Results

There were 27 participants, 8 (29.6%) males and 19 (70.4%) females, with a median age of 32 years (range 20-58). They included 21 (77.8%) from Accra, 4 (14.8%) from Kumasi, and 2 (7.4%) from Tema. These participants experienced the lockdown between 7 and 21 days, median days 21 (see Table 1 for the rest), thus, an indication that some individuals moved out of or into the lockdown cities during the period. Furthermore, we analyzed and presented the results based on our pre-determined themes, and immerging sub-themes such as PA (exercise, physical inactivity, and space for exercising), eating habits (positive eating and negative eating), and boredom and weight gain. The themes and their sub-themes and the quotes are presented in Table 2 and Table 3.

**Table 1:** characteristics of participants

Characteristics	N/(%) or mean	Median (range)
**Age**	32	32(20-58)
**No. of days in lockdown**	21	21 (7-21)
**Gender**		
Male	8(29.6%)	
Female	19(70.4%)	
**Education**		
Postgraduate	12(44.4%)	
Undergraduate	14(51.9%)	
Senior high school	1(3.7)	
**Location**		
Accra	21(77.8%)	
Kumasi	4(14.8%)	
Tema	2(7.4%)	
**Employment**		
Accounting and finance	1(3.7%)	
Business and administration	3(11.1%)	
Education sector worker	6(22.3%)	
Healthcare worker	7(25.9%)	
Mechanical engineer	1(3.7%)	
Minister of religion	1(3.7%)	
Publishing	1(3.7%)	
Sports administrator	1(3.7%)	
Student	4(14.8%)	
Writer	1(3.7&)	
Unemployed	1(3.7%)	

**Table 2:** physical activity themes identified from the respondents´ transcripts

Themes	Sub-themes	Quotations
**Physical activity**	**Exercise**	...“ So I have started exercising and this is day 7”.
**-**	**-**	“Dancing sometimes”
**-**	**-**	“But I did some indoor exercises to keep fit”
**-**	**-**	“I try as much as possible to exercise to remain active”.
**-**	**-**	“Increase response to exercises” “try to exercise”
**-**	**-**	“Not much affected because we moved around in the house, doing other things”
**-**	**-**	“I'm already used to such things but I exercise once daily to reduce the already gained weight”
**-**	**-**	“I do have a stroll in the evening...”
**-**	**-**	“We do physical exercise indoors”
**-**	**-**	“We play games together”
**-**	**-**	“We did a lot of physical exercise”
**-**	**-**	“I had to seriously reduce the daily meal because I didn't know when the lockdown would end”
**-**	**Physical inactivity**	“Had less physical exercises”.
**-**	**-**	“It was very tiring doing nothing, no exercise throughout the day so I decided to get myself busy with reading”
**-**	**Lack of space for exercise**	“Generally, we don't have enough space and so there was little physical exercise so I gained wait”
**-**	**-**	“It is very bad, not having enough place to walk”
**-**	**-**	“Hmmm, nowhere to do any exercise even if I wanted to do something, look, the whole community no pack, no space for anything, bad”

**Table 3:** eating patterns, boredom and weight gain among the respondents

Themes	Sub-themes	Quotations
**Eating behavior**	**Positive eating**	“Tried to limit eating since being inactive was more”
		“I eat a balanced meal when I can”
		“Eating habits are normal...I eat at most 3 times daily”
		“...I was eating well, with normal weight”
		“...Changed our eating habit by not eating too much so that we wouldn't gain weight unnecessarily after the lockdown”
	**Poor eating**	“Eating habit was bad a little”
		“Appetite has increased, I gained some weight”
		“Initially I kept eating anyhow but that reduce a bit, oh, I gained much weight”
		“I ate like a baby, always putting something into the mouth, until a blot”
	**Boredom and weight gain**	“I feel bored and gaining weight”
		“I gained more weight”
		“Fewer interactions with colleagues due to remote working contributes to boredom at times”
		“It was like being in prison, caged, nowhere to go, everywhere you turn, police”
		“so much boredom and gained some weight”
		“My life during the lockdown is not too different from my regular life with regards to my social life and going out. The only difference now is that I don't have the option of stepping out, even once in a while. And that can be a little frustrating”

## Discussion

This study aimed to qualitatively analyze the experiences (PA, eating behavior, boredom, and weight gain) of individuals in Accra, Tema, and Greater Kumasi who experienced the 21-day lockdown as COVID-19 pandemic containment measure in Ghana. The findings revealed that majority of the respondents spent 21 days under the lockdown but few others also experienced it for about seven days. Moreover, during the lockdown period, some individuals attempted to increase their PA levels doing exercises, dancing but others also experienced a tremendous degree of inactivity because of lack of space. We further observed that the eating habit of the residents changed, where many tried to eat healthily but others experienced poor dieting behavior. Besides, boredom set in for some of the residents who also reported weight gain as a result of the lockdown.

The focus of the lockdown was to restrict the movement of people to contain the spread of the COVID-19 virus in Accra, Tema, and Greater Kumasi, and Ghana at large [4]. However, this purpose could be defeated as some people spent just seven days under lockdown, an indication that during the period, people moved between the cities under the lockdown and other cities not under the lockdown. Thus, persons moving out of the lockdown cities could carry the infection to other cities or those moving in could get infected with the virus in the lockdown cities, and that, such inter-city movement during the period of lockdown could probably increase the cases of COVID-19 infection. Few persons tried to keep up their PA levels during the lockdown era in Ghana but others reported sedentariness due to lack of or inadequate space. For example, WHO Europe [16] encourages individuals under quarantine to engage in PA but also stressed the availability of space for such important health behavior. Thus, keeping up with PA during this time of COVID-19 pandemic and associated lockdown does not only reduce psychological health challenges such as anxiety, boredom, and depression, that come with the virus, but will promote the immune response system to limit the effects of the virus [20].

Furthermore, family PA may promote the socio-emotional health of individual family members which further increases personal immunity against the COVID-19 virus. The physical inactivity reported by the participants calls for more PA education since increase sedentary behavior could further compromise personal immunity and the general health state of the individual [7]. Also, community and residential accommodation spaces are required if home PA and more activeness are to be encouraged because many of the PHPMs to curtail the spread of COVID-19 pandemic are creating obesogenic environments [12]. Coupled with boredom (a psychological state that can lower immunity), the lockdown increased poor eating behavior of individuals, resulting in weight gain. Though few participants reported self-consciousness in eating, a lot more believed their eating habits during the lockdown was inappropriate and increased their weight at the end of the period. We argue that the COVID-19 induced lockdown has the tendency to produce increasing physical inactivity and boredom, situations that are also promoting poor eating behavior among affected individuals [13]. These “triple dragons” (physical inactivity, boredom, and poor eating behavior) will result in weight gain, as reported by the participants. Physical inactivity, poor eating behavior, and the feeling of boredom could have adverse health effects such as increased obesity, and other closely related chronic illnesses i.e. diabetes, hypertension, and heart diseases on individuals [15]. Thus, in the quest to fight the COVID-19 pandemic, Ghana may live to fight obesity pandemic and other related illnesses soon if PA life of its citizen is not promoted.

## Conclusion

The aim of this study was to qualitatively analyze PA, inactivity, eating behavior, boredom, and weight gain as experienced by individuals in Accra, Tema, and Greater Kumasi who lived under the 21-day lockdown as COVID-19 pandemic containment measure in Ghana. The lockdown of Accra, Tema, and Greater Kumasi may not achieve its intended purposes because few people moved across cities under the lockdown and others (not under the lockdown) during the period of COVID-19 induced lockdown. This situation that could spread the virus further, and defeat the intended purpose of the lockdown. Subsequently, the government of Ghana and/or local authorities would want to be stricter to prevent movements of people in-and-out of lockdown city(s) if the COVID-19 pandemic is to be contained. Though some people tried to do some level of PA, many more were inactive, a situation some attributed to lack of space. Coupled with physical inactivity, many felt bored, engaged in poor eating behaviors, and gained more weight (COVID-19 induced weight gain). Therefore, Ghana may be fighting another epidemic such as COVID-19 induced obesity and its closely related chronic illnesses if pragmatic efforts are not rolled out or incorporated into public health education campaigns aimed at combating the COVID-19 pandemic. Thus, the Ghana Health Service and other health and sport-related Non-Governmental Organizations need to urgently develop evidence-based health education to address PA promotion, “good” eating behavior, and means of dealing with boredom during and after lockdown period. As a country and individuals, we would need to rethink of designing and redesigning our communities and homes to create enough PA space, and also educate citizens on how to use limited space for PA. Beyond these, there is the need for individual researchers and institutions to objectively measure PA, sedentariness, eating behaviors, and the obesity of persons in Accra, Tema, and the Greater Kumasi. This study identified that persons who lived under the lockdown in Accra, Tema, and Greater during the COVID-19 induced lockdown experienced some negative changes, including physical inactivity, poor eating behaviors, feeling of boredom, and gained weight. However, we acknowledge that in collecting the data, thematic redundancy might have been achieved before the 27^th^ respondent, but we were not able to determine that because of the online data collection method used. This poses a limitation to the study and is acknowledged.

### What is known about this topic

- Physical activity participation promotes healthy life including controlled weight and reduced propensity of obesity;
- Sedentariness and poor dietary behaviors are factors to obesity, diabetes and other metabolic diseases, cardiovascular diseases, and even some psychological and emotional health challenges;
- Restrictive movement of people creates physical, psychological and emotional health challenges.

### What this study adds

- Some people move between cities under the lockdown and others, which may increase the likelihood of the spread of the COVID-19 virus, and defeat the purpose of the lockdown;
- COVID-19 induced lockdown increases the chances for sedentariness and poor eating habit among some persons in Accra, Tema, and Greater Kumasi; COVID-19 induced lockdown created boredom for the persons in Accra, Tema, and Greater Kumasi;
- Some persons in Accra, Tema, and Greater Kumasi lack available space for engaging in the needed physical activity during the period of lockdown; many people gained weight during the lockdown probably as a result of boredom, poor eating and physical inactivity.
